# A Case of Occlusion of Steno’s Ducts

**Published:** 1891-02

**Authors:** 


### A Case of Occlusion of Steno's Ducts.

In the *Medical Record*, November 29th, Dr. B. C. Loveland reports an interesting case of occlusion of Steno's Ducts. The patient, a lady of middle age, had been troubled with a dry mouth since she had diptheria twelve or thirteen years ago. Dr. Loveland described the appearance of the mouth as follows:

"Her mouth was indeed dry, for, as I looked into it there seemed only a sticky mucus about the teeth and under the tongue. And when I took hold of her lips and cheek to look for the openings of Steno's ducts, the mucous membrane wrinkled like dry tissue-paper does when stretched. I could not see at first the openings of the ducts, and could not press any saliva out. But when she attempted to eat, the parotid glands would swell up so as to make her look like a patient with the mumps."

The ducts were opened by introducing a small sized probe, Bowman's No. 1, and subsequently using larger sized probes, until a No. 8 Bowman probe could be used freely, the treatment being given every day for a while, then every other day, and then once a week, the length of time from first treatment to close was from March till June.

Dr. Loveland further describes the case:

"It was a long time after the ducts were well opened before the salvia took on a normal appearance, for at first it was about like the white of an egg, and had to be pressed out. It also contained some pus. She assured me that she had been unable to chew solid food, and had lived almost entirely on liquids, for more than a year and a half before I saw her, and had much discomfort even in taking liquid food, on account of the swelling and pain caused by the secretion of salvia, which could not find exit and had to be taken back into the tissues. She had considerable trouble with the lymphatics of the neck as a result."

Dr. Loveland states that the strictures in the ducts were probably the result of diptheria imflammation.

